# The furosemide stress test: Perspectives for acute kidney injury diagnosis

**DOI:** 10.1590/2175-8239-JBN-2021-E010

**Published:** 2021-10-27

**Authors:** Francisco J. López Hernández

**Affiliations:** 1 Instituto de Investigación Biomédica de Salamanca, Grupo de Investigación Traslacional en Enfermedades Renales y Cardiovasculares, Salamanca, España.; 2 Fundación Instituto de Estudios de Ciencias de la Salud de Castilla y León, Soria, España.; 3 Universidad de Salamanca, Departamento de Fisiología y Farmacología, Salamanca, España.; 4 Instituto de Salud Carlos III, National Network for Kidney Research REDINREN, RD016/0009/0025, Madrid, Spain.; 5 Grupo de investigación en Biomedicina en Cuidados Críticos, Valladolid, España.

Acute kidney injury (AKI) is a poorly defined syndrome characterized by sudden excretory dysfunction. AKI has a high and increasing incidence, a high mortality rate, especially in critically ill patients, and serious medium- and long-term consequences, including progression to chronic kidney disease and elevated cardiovascular morbimortality^1^. AKI severity is associated with worsened outcomes. As AKI can evolve very rapidly and the available interventional arsenal is essentially limited to eliminating (or treating) the cause and maintaining hydration, the earliest possible diagnosis is critical for optimized clinical management^2^. For similar reasons, an ideal diagnosis should include prognostic estimations. However, no factors other than severity have been clearly associated with outcome. Furthermore, the current diagnostic criteria, namely the creatinine-based international scoring scales, such as RIFLE, AKIN and KDIGO, only allow a late classification of severity^3^.

The creatinine-centered view of AKI neglects the etiopathologic granularity required for personalized diagnosis. Because of the heterogeneity of AKI and the complexity of the underlying biological processes, this limitation is inherent to all diagnostic methods based on a single parameter or biomarker. Accordingly, new (mostly urinary) biomarkers have emerged in the last two decades that capture pathophysiological information missed by creatinine testing, with earlier sensibility, purportedly complementing diagnosis^4^. However, an important limitation to their use in routine diagnostic procedures and definitions is the uncertainty about their biological and pathophysiological significance^5^. The exact processes leading to the appearance of these biomarkers in various biological samples are not completely understood^6^. Thus, their potential clinical utility derives only from statistical associations between biomarker levels and AKI outcomes at the population level. It is thus necessary to develop multiparametric diagnostic methods that include biomarkers with defined pathophysiological significance. In addition to its deep-rooted tradition, this is probably one of the reasons why creatinine has been retained as a gold standard of renal function in nephrology: the fact that, despite suboptimal specificity, we know that creatinine is a surrogate for glomerular filtration rate (GFR). A related problem is the historically habitual reduction of renal function evaluation to GFR status.

Recently, the furosemide stress test (FST) has gained increasing attention as a potential candidate for the evaluation of tubular functionality. The FST was rescued a few years ago and standardized for potential nephrologic diagnosis^7^^,^ 8 as an application of previous observations^9^^,^ 10. The FST detects subclinical tubular alterations reflected in the abnormal diuretic response to a single dose of furosemide with high sensitivity but low resolution capacity^11^. In fact, intact tubules are necessary to ensure a normal diuretic response to furosemide, so that alterations in virtually all segments of the nephron could theoretically alter the result of the FST. This makes the FST a double-edged sword with great multivalence at the expense of low specificity. In addition, adaptive and compensatory responses along the nephron in response to evolving conditions (such as electrolyte overloads)^12^ may occasionally alter the response to the FST, leading to false positives and negatives.

In this issue of JBN, Pon et al.^13^ report a study extending and corroborating the initial observations by Chawla et al.^8^ to a population in the intensive care setting in India. In both studies, the FST results in early stage AKI patients (KDIGO 1 and 2) predict progression to KDIGO stage 3 with reasonably high accuracy. This last statement is important. High accuracy means that there are a number of false positives and false negatives in this study that are misclassified as AKI progression by the FST. In other words, some patients with altered FST response did not progress to stage 3, and some patients who progressed had normal FST response, respectively. Clearly, there are unknown additional factors that determine AKI progression either alone or in mandatory combination with the alterations detected by the FST. For example, some purely pre-renal AKI patients in stages 1 and 2 would be expected to progress to stage 3. Given the hemodynamic nature of pre-renal AKI and the parenchymal nature of the alterations detected by the FST, it would be impossible for this test to predict the behavior of these patients. Accordingly, the predictive accuracy of this test (and virtually all single-parameter tests) is highly dependent on the characteristics of the study population, which may potentially vary from population to population, and hence from study to study. This reinforces the limitations of a single parameter in describing the behavior of etiopathologically heterogeneous populations and the need to combine multiple parameters to further stratify individuals, as performed by Blanco-Gozalo et al.^14^

Overall, the FST provides a prospective diagnostic parameter that contains some defined pathophysiological knowledge and complementary creatinine-insensitive information, but it needs further substantial contextualization. Furthermore, as a stress test, the FST fits perfectly with the strengthening concept of an acquired predisposition to AKI caused by a reduced functional reserve^15^. In line with the reductionist concept of renal function, before the advent of the FST, a decreased functional reserve referred exclusively to the reserve of GFR, known as *renal functional reserve*. The FST widens the concept of functional reserve to the tubular compartment, while highlighting the need to revise the nomenclature and ontology behind the term.

## References

[1] Ronco C, Bellomo R, Kellum JA (2019). Acute kidney injury. Lancet.

[2] Soni SS, Ronco C, Katz N, Cruz DN (2009). Early diagnosis of acute kidney injury: the promise of novel biomarkers. Blood Purif.

[3] Vanmassenhove J, Biesen WV, Vanholder R, Lameire N (2019). Subclinical AKI: ready for primetime in clinical practice?. J Nephrol.

[4] Kulvichit W, Kellum JA, Srisawat N (2021). Biomarkers in Acute Kidney Injury. Crit Care Clin.

[5] Yang SY, Chiou TT, Shiao CC, Lin HY, Chan MJ, Wu CH, Sun CY, Wang WJ, Huang YT, Wu VC, Chen YC, Fang JT, Hwang SJ, Pan HC (2021). Nomenclature and diagnostic criteria for acute kidney injury - 2020 consensus of the Taiwan AKI-task force. J Formos Med Assoc.

[6] Sancho-Martínez SM, Blanco-Gozalo V, Quiros Y, Prieto-García L, Montero-Gómez MJ, Docherty NG, Martínez-Salgado C, Morales AI, López-Novoa JM, López-Hernández FJ (2020). Impaired Tubular Reabsorption Is the Main Mechanism Explaining Increases in Urinary NGAL Excretion Following Acute Kidney Injury in Rats. Toxicol Sci.

[7] Chawla LS, Davison DL, Brasha-Mitchell E, Koyner JL, Arthur JM, Shaw AD, Tumlin JA, Trevino SA, Kimmel PL, Seneff MG (2013). Development and standardization of a furosemide stress test to predict the severity of acute kidney injury. Crit Care.

[8] Rewa OG, Bagshaw SM, Wang X, Wald R, Smith O, Shapiro J, McMahon B, Liu KD, Trevino SA, Chawla LS, Koyner JL (2019). The furosemide stress test for prediction of worsening acute kidney injury in critically ill patients: A multicenter, prospective, observational study. J Crit Care.

[9] Baek SM, Brown RS, Shoemaker WC (1973). Early prediction of acute renal failure and recovery. II. Renal function response to furosemide. Ann Surg.

[10] Baek SM, Brown RS, Shoemaker WC (1973). Early prediction of acute renal failure and recovery. I. Sequential measurements of free water clearance. Ann Surg.

[11] Casanova AG, Fuentes-Calvo I, Hernández-Sánchez MT, Quintero M, Toral P, Caballero MT, Martínez-Salgado C, Morales AI, Layton AT, Eleno N, López-Hernández FJ (2021). The furosemide stress test and computational modeling identify renal damage sites associated with predisposition to acute kidney injury in rats. Transl Res.

[12] Jensen IS, Larsen CK, Leipziger J, Sørensen MV (2016). Na(+) dependence of K(+) -induced natriuresis, kaliuresis and Na(+) /Cl(-) cotransporter dephosphorylation. Acta Physiol (Oxf).

[13] Pon AG, Vairakkani R, Mervin EF, Srinivasaprasad ND, Kaliaperumal T (2021). Clinical significance of frusemide stress test in predicting the severity of acute kidney injury. Braz. J. Nephrol..

[14] Blanco-Gozalo V, Casanova AG, Sancho-Martínez SM, Prieto M, Quiros Y, Morales AI, Martínez-Salgado C, Agüeros-Blanco C, Benito-Hernández A, Ramos-Barron MA, Gómez-Alamillo C, Arias M, López-Hernández FJ (2020). Combined use of GM2AP and TCP1-eta urinary levels predicts recovery from intrinsic acute kidney injury. Sci Rep.

[15] Sharma A, Mucino MJ, Ronco C (2014). Renal functional reserve and renal recovery after acute kidney injury. Nephron Clin Pract.

